# The Merriment of Mr. Stephen Coleridge

**Published:** 1915-06-12

**Authors:** 


### THE MERRIMENT OF MR. STEPHEN COLERIDGE.

Mr. Stephen Coleridge has often presented himself as an instructor of his fellows. He is well known, too, as a champion who endeavours "to prove his doctrine orthodox by apostolic blows and knocks." His neck is clothed with thunder and he snuffeth the battle from afar. For our own part we have often felt that his devotion to the cause he has espoused merits some measure of admiration, and may even be quoted as an example to souls of a more languid order. No doubt we think he is wrong in his aim, and we find it difficult at times to respect his methods. But we willingly recognise his right to fight the anti-vivisectionist battle, since his convictions justify it, and we would claim for him exactly the same measure of fair play as we would ask for ourselves. With his recent and renewed presentation of himself as the victim of "frantic prejudice and malignant misrepresentation" we are not concerned. In the *rôle* of the hero leading the fight he has some attractive qualities, but the part of the oppressed and virtuous sufferer does not become him. It is in armour, not in tears, that we like him best.

Lately it seems that, departing for the time being from the heroic and the pathetic, Mr. Coleridge has endeavoured to cultivate a comic vein. And here a terrible misfortune has befallen him. For the world in general, and ourselves in particular, have failed to be amused. The author of a joke which misses fire, if he is a prudent man, usually says nothing about it; he contents himself with internal reflections on the stupidity of his audience. Such, however, is not Mr. Coleridge's way. Our readers may not know it, but nevertheless it is true that he is the editor of what he would call a "little sheet," bearing a title too ponderous here to quote. In this he tells us of his joke and of the merriment and laughter which it has excited—in himself. Others, it seems, have not laughed, and so once again Mr. Coleridge falls back upon the "Ercles' vein." Had he condemned us all for lack of humour we might have denied the impeachment, but at least we could, in the circumstances, have understood the charge. So obvious an explanation he refuses. The real cause of our failure to join in the "merriment" is, it seems, our knowledge that he is "opposed to vivisection." Frankly, we admit that we have heard of this opposition. But we were quite unaware that this knowledge had had a paralysing effect on any of our faculties. Rather we had numbered it with the placid and monotonous announcement which records the death of Queen Anne. It is, we quite admit, very annoying, even to the regenerate, to be the begetter of an abortive joke. But Mr. Coleridge must try again, and perhaps the next one will have more vitality. The circumstances in which the joke was still-born merit just a word of attention. Mr. Coleridge recognises with a fine air of detachment that the anti-typhoid inoculation controversy can only be settled by "unbiassed statistics," and he expresses no opinion on the matter. But he knows that, among others, Sir Almroth Wright is immersed in the study of this and allied questions which are vital to the health and lives of our soldiers. Further, he has discovered that once upon a time Sir Almroth wrote or said something about dirt and disease, and he has served this up to his own infinite amusement. It has nothing on earth to do with vaccines or inoculations, but the announcement of it when its author has on hand matters more important than newspaper controversy may help to undermine confidence in the military medical service. This is the occasion on which Mr. Coleridge, to quote his own journal, has enjoyed "a good laugh," and we have failed to join in the merriment. Perhaps, after all, the joke is a good one, and it is the times which are unpropitious. When peace returns Mr. Coleridge may resume the comic *rôle* with better prospect of success, and we shall await his performance with interest.

